# Post-transplant lymphoproliferative disorder (PTLD): single institutional experience of 141 patients

**DOI:** 10.1186/s40164-017-0087-0

**Published:** 2017-09-29

**Authors:** Rohit Bishnoi, Ravneet Bajwa, Aaron J. Franke, William Paul Skelton, Yu Wang, Niraj M. Patel, William Birdsall Slayton, Fei Zou, Nam H. Dang

**Affiliations:** 1 Department of Medicine, University of Florida, PO Box 100238, Gainesville, FL 32610-0238 USA; 2 Department of Medicine, University of Florida, PO Box 100278, 1600 SW Archer Rd, Gainesville, FL 32610 USA; 3 Department of Medicine, University of Florida, PO Box 100277, Gainesville, FL 32610-0277 USA; 4 Department of Biostatistics, University of Florida, PO Box 117450, Gainesville, FL 32611 USA; 5 Division of Pediatric Hematology/Oncology, University of Florida, PO Box 100298, Gainesville, FL 32610-0298 USA

**Keywords:** PTLD, Post-transplant lymphoproliferative disorder, Prognostic factors for PTLD, EBV PTLD, Rituximab for PTLD

## Abstract

**Background:**

Post-transplant lymphoproliferative disorder is a well-recognized but rare complication of hematopoietic stem cell and solid organ transplant. Due to rarity of this disease, retrospective studies from major transplant centers has been the main source to provide treatment guidelines, which are still in evolution. The sample size of this study is among one of the largest study on PTLD till date reported throughout the world.

**Methods:**

This study was performed at University of Florida which is one of the largest transplant center in South East United States. We performed treatment and survival analysis along with univariate and multivariate analysis to identify prognostic factors.

**Results:**

We reviewed 141 patients diagnosed with PTLD over last 22 years with median follow-up of 2.4 years. The estimated median overall survival of the entire group was 15.0 years. Sub group analysis showed that 5-year overall survival rates of pediatric population were 88% (median not reached). For adults, median OS was 5.35 years while for elderly patients it was 1.32 years. The estimated median OS of patients with monomorphic PTLD was 9.0 years while in polymorphic PTLD was 19.3 years. Univariate analysis identified gender, age at transplant and PTLD diagnosis, performance status, IPI score, allograft type, recipient EBV status, multiple acute rejections prior to PTLD diagnosis, PTLD sub-type, extra-nodal site involvement, immunosuppressive drug regimen at diagnosis, initial treatment best response were statistically significant prognostic factors (p < 0.05). On multivariate analysis, age at PTLD diagnosis, recipient EBV status, bone marrow involvement, and initial best response were statistically significant prognostic factors (p < 0.05). Surprisingly, use of Rituximab alone as upfront therapy had poor hazard ratio in the cumulative group as well less aggressive PTLD subgroup comprising of early lesions and polymorphic PTLD.

**Conclusions:**

Our experience with treatment and analysis of outcomes does challenge current role of Rituximab use in treatment of PTLD. Currently as we define role of immunotherapy in cancer treatment, the role of acute rejections and immunosuppressant in PTLD becomes more relevant as noticed in our study. This study was also able to find new prognostic factors and also verified other known prognostic factors.

## Background

Post-transplant lymphoproliferative disorder (PTLD) is a well-recognized complication of Hematopoietic Stem Cell Transplant (HSCT) and Solid Organ Transplant (SOT). It was first described in renal transplant recipients in 1968 by Doak et al. [1] and the term PTLD was first introduced in 1984 by Starzl et al. [2]. PTLD was classified as a separate entity for the first time in 2008 World Health Organization (WHO) classification of lymphoma [3].

The reported incidence of PTLD varies in different transplant centers likely secondary to different immunosuppressive regimens, allograft types and patient population characteristics. PTLD following SOT occurs in up to 20% with the highest incidence in intestinal and multi-organ transplants (5–20%), followed by lung and heart transplants (2–10%) and then by renal and liver transplants (1–5%) [4]. Higher incidence of PTLD in heart, lung, intestinal and multi-organ transplants has been attributed to greater immunosuppression necessary to protect allografts in these patients [5–7]. In the pediatric population with SOT, PTLD incidence varies from 2.2% in the kidney to 15% in lung transplant recipients [8]. In contrast, PTLD incidence is about 4% after hematopoietic stem cell transplant (HSCT) [9]. Historically, mortality rates in SOT-related PTLD were 50–70% and up to 70–90% in HSCT, although recent data suggests that outcomes have been improving and comparable to SOT [10, 11].

In the updated 2016 WHO lymphoma classification, PTLD has been subclassified as Plasmacytic hyperplasia PTLD, Infectious mononucleosis PTLD, Florid follicular hyperplasia PTLD, Polymorphic PTLD, Monomorphic PTLD (B- and T-/NK-cell types) and Classical Hodgkin lymphoma PTLD [12]. Diffuse large B-cell lymphoma (DLBCL) accounts for the majority of the cases of PTLD. Most PTLD cases are of B-cell origin while 5–10% can be T/NK cell or Hodgkin lymphoma type. About 70% of cases of PTLD is associated with Epstein-Barr virus (EBV), especially in cases which occur early after transplantation. Pathogenesis of the disease is linked to EBV proliferation in the setting of chronic immunosuppression leading to an inhibition of T cells immune function. However, in 30% of EBV-negative PTLD patients, pathogenesis is not clearly understood.

The primary goal of PTLD treatment is cure while retaining allograft function. Historically, reduction in immunosuppression (RIS) has been the primary mode of treatment. Current guidelines recommend RIS at lowest tolerated levels based on PTLD type, stage, critical illness and allograft risk and then monitoring closely for response and escalation of therapy in the case that RIS alone is not sufficient [13]. Efficacy of this modality depends on PTLD sub-types, disease extent and other prognostic markers [14]. Furthermore, the durability of response by RIS alone is maintained only in about 10% of the patients [2, 15].

Choquet et al. conducted the first prospective (Phase II) trial of single-agent rituximab in SOT-related PTLD after the failure of RI [16]. At 1 year, the Overall Response Rate (ORR) was 34%, and Overall Survival (OS) rate was 67%. When applicable, single agent rituximab is now considered as the first option in low-risk or patients failing RIS. The initial response to rituximab alone as induction therapy is also noted to be a prognostic factor for PTLD [17]. Due to the toxicity of chemotherapy, it is usually reserved for patients with high-risk, aggressive disease or who failed modalities above including RIS and/or single agent rituximab [17, 18]. In adults, anthracycline-based chemotherapy regimens have shown good success for PTLD, and R-CHOP (rituximab, cyclophosphamide, doxorubicin, vincristine, and prednisolone) is currently the preferred regimen for B-cell PTLD with ORR up to 90% [19, 20]. In the pediatric population, the chemotherapy regimen RCP (rituximab, cyclophosphamide, and prednisone) has shown a good response and is preferred regimen [21, 22].

Due to the rarity of this disease, different transplant centers have reported incidence and outcomes and have identified several factors affecting prognosis. As University of Florida (UF) is one of the largest transplant centers in the southeastern United States, we here report our experience with PTLD which we expect will help in better understanding of this rare entity.

## Methods

### Study population and data collection

The study population was identified mainly from the patient database at UF Health using ICD 9/10 codes and also from United Network for Organ Sharing (UNOS). The studied patient population comprised of all patients including both pediatric and adults, with pathologically confirmed PTLD after SOT or HSCT.

The primary source of data collection was individual chart review by the study team, and no patient was contacted directly or indirectly for data collection. Data were collected in a Health Insurance Portability and Accountability Act (HIPAA) compliant data tool, Research Electronic Data Capture (REDCap) provided by UF. Data was collected for demographics, transplant and immunosuppression history, EBV and CMV serostatus, diagnosis, treatment and outcomes of PTLD.

### Data interpretation and statistical analysis

Data were analyzed using SAS software 9.4 (SAS Institute, Cary, NC.) with the help of the UF department of biostatistics. Our primary outcome was overall survival (OS). A secondary analysis was then performed to identify factors affecting OS. The hazard ratio (HR) was calculated, and univariate and multivariate analysis was done. Disease outcome was measured as complete remission (CR), partial remission (PR), stable disease (SD) and progressive disease (PD). Disease control rate (DCR) and overall response rates (ORR) were also calculated. DCR was defined as the sum of CR, PR, and SD while ORR was defined as the sum of CR and PR.

## Results

A total of 151 patients were identified as per study inclusion criteria. Patients with very limited records were excluded (n = 10), and so 141 patients were included for final analysis. These patients were diagnosed with PTLD over a period of 22.2 years ranging from September 1994 to December 2016. These patients were transplanted as early as November 1988 to as late as May 2016. Data were collected up to Jan 31, 2017, of all eligible patients. Median follow up time from PTLD diagnosis was 2.4 years.

### Estimated overall survival, univariate and multivariate analysis

**Fig. 1:**
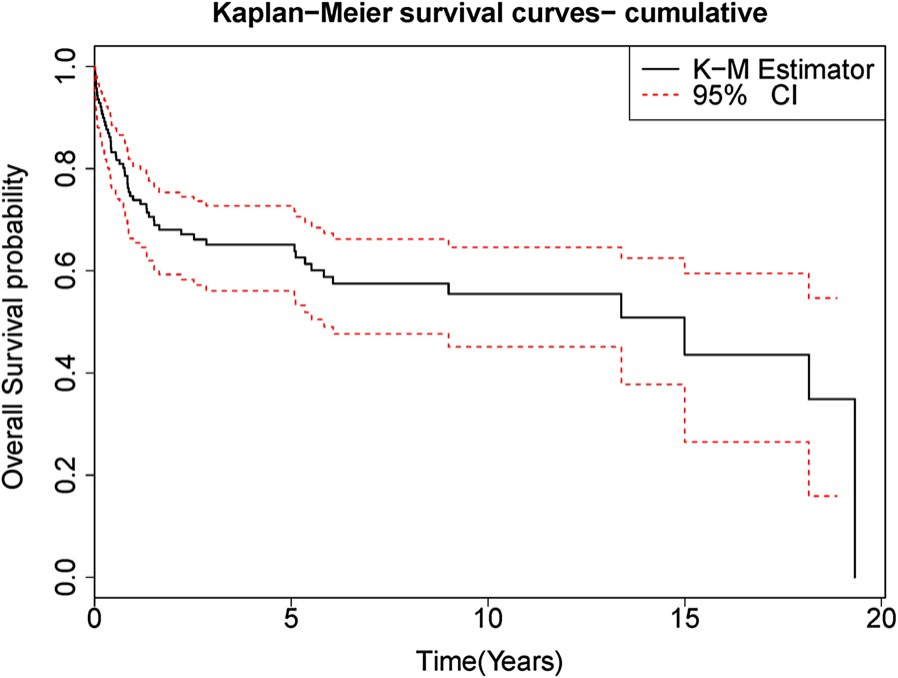
KM estimate of overall survival of cumulative group

The estimated median OS of the entire group was 15.0 years (CI 5.83–19.33), see Fig. 1. For pediatric population median was not reached but 5 year survival rates were 88%, in adults estimated median OS was 5.35 years (CI 1.33–18.15) while for elderly patients estimated median OS was 1.32 years (CI 0.34–2.84). The estimated median OS in patients who achieved CR was 19.3 (CI 18.15–19.33) years while the estimated median OS in patients who achieved PR was 5.4 years (CI 0.17–5.35). The estimated median OS of patients with monomorphic PTLD was 9.0 years (CI 2.52–NR) while in polymorphic PTLD was 19.3 years (CI 1.32–19.32).

**Fig. 2:**
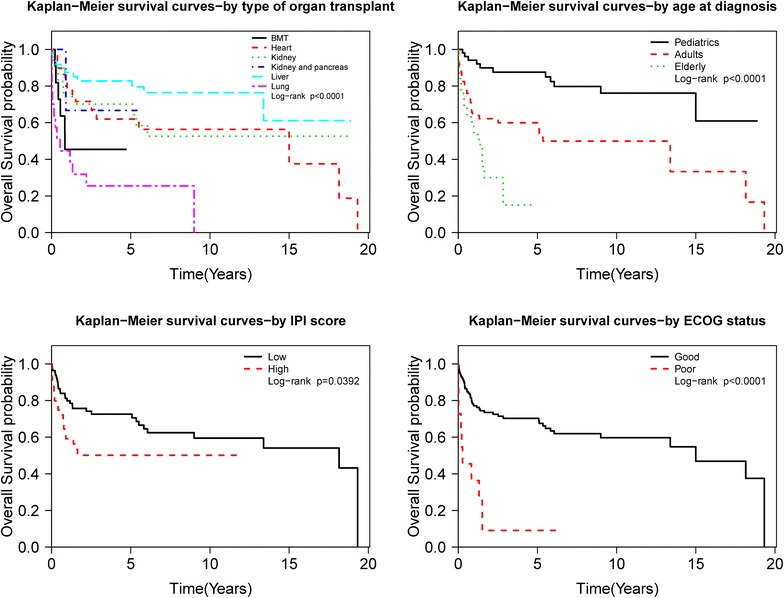
KM estimate based on significant factors

**Fig. 3:**
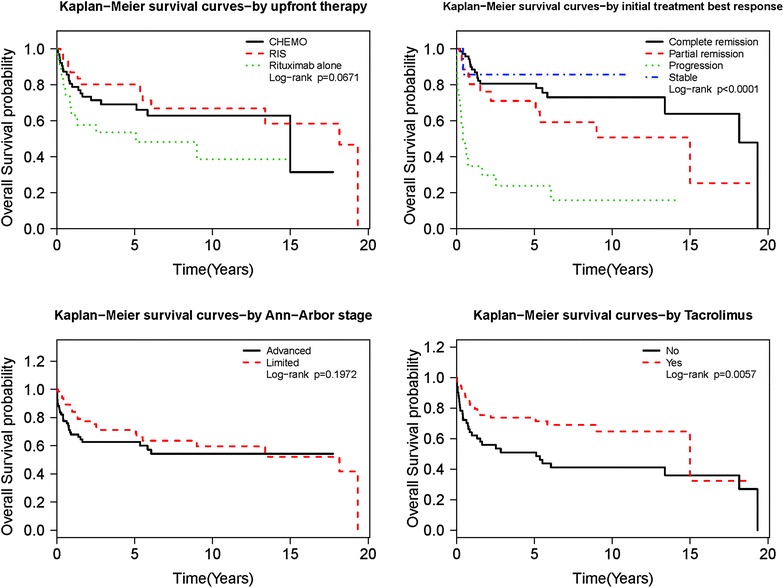
KM estimate based on significant factors

On univariate analysis for OS, we found that patient gender, age at transplant and PTLD diagnosis, performance status, IPI score, allograft type, recipient EBV status, multiple acute rejections prior to PTLD diagnosis, PTLD sub-type, extra-nodal site, immunosuppressive drug regimen at diagnosis, initial treatment best response were statistically significant prognostic factors (p < 0.05). On multivariate analysis age at diagnosis, recipient EBV status, bone marrow involvement, and initial best response were statistically significant prognostic factors (p < 0.05). Figures 2 and 3 shows KM analysis of other significant prognostic markers.

### Demographics

Demographic profile of these 141 patients showed that 62.4% were males (n = 88) and 37.6% were females (n = 53). Racial distribution showed that 71.6% were Whites (n = 101) followed by 17.7% of Blacks or African Americans (n = 25) and rest others. 12.8% (n = 18) had a past medical history of malignancy before transplant. The median age at the time of PTLD diagnosis was 41.6 years (Range 1.8–79.4 years). Of these, the pediatric (< 18 years) population consisted of 38.3% (n = 54) while 61.7% (n = 87) were adults. Of the 141 patients, 16.3% patients (n = 23) were aged 65 years or above.

#### Prognostic impact

Our study revealed female gender as a better prognostic marker with HR of 0.553 (p: 0.0427, CI 0.311–0.981) while race and ethnicity were not statistically significant. A past medical history of malignancy showed a trend towards poorer prognostic marker but was not statistically significant.

Age at PTLD diagnosis was also statistically significant with pediatric patients having HR of 0.241 (p < 0.0001, CI 0.123–0.473) and elderly patients with HR of 3.543 (p < 0.0001, CI 1.894–6.628).

### Transplant history

Median age at transplant was 33.3 years (range 1 month to 68 years). Median time from transplant to PTLD diagnosis was 4.1 years (range 1 month to 18.4 years). Type of organ transplant distribution is as detailed in Table 1. Of 17 lung transplant patients 64.7% (n = 11) had single lung transplant while rest 35.3% (n = 6) patients had a double lung transplant.

**Table 1:** Study population characteristics with univariate analysis

Variables	Sub-types	*n*	Percentage	p-value	Hazard ratios	95% confidence limits
Gender	Males	88	62.4			
	Females	53	37.6	*0.0427*	0.553	0.311–0.981
Race	White	101	71.6	0.1314	1.670	0.858–3.252
	Black or African American	25	17.7	0.8428	0.930	0.452–1.911
	Asian	4	2.8	0.5190	0.521	0.072–3.776
	Native Hawaiian or other Pacific Islander	1	0.7	0.9862	0.000	0.000–0.000
	Other/Unknown	10	7.1	0.1292	0.216	0.030–1.564
Transplant organ	Liver	49	34.8	*0.0023*	0.357	0.184–0.693
	Kidney	31	22.0	0.9348	0.973	0.502–1.886
	Heart	30	21.3	0.9153	0.967	0.517–1.809
	Lung	17	12.1	*< .0001*	4.380	2.327–8.245
	Kidney and pancreas	3	2.1	0.8666	0.844	0.116–6.116
	BMT	11	7.8	0.0746	2.197	0.925–5.219
Age group at transplant	Pediatrics (< 18 years)	58	41.1	*< .0001*	0.207	0.105–0.407
	Adults (18–64 years)	74	52.5	*< .0001*	3.316	1.836–5.990
	Elderly (> 65 years)	9	6.4	*0.0472*	2.573	1.012–6.545
Age group at PTLD diagnosis	Pediatrics (< 18 years)	54	38.3	*< .0001*	0.241	0.123–0.473
	Adults (18–64 years)	64	45.4	0.0633	1.657	0.972–2.823
	Elderly (> 65 years)	23	16.3	*< .0001*	3.543	1.894–6.628
PTLD onset time	Early (less than 1 year)	35	24.8	0.8426	0.939	0.502–1.754
	Late (after 1 year)	73	51.8	0.7821	0.928	0.546–1.577
	Very late (after 10 years)	33	23.4	0.5787	1.196	0.636–2.246
Recipient serostatus at transplant	EBV positive	82	58.2	*0.0127*	2.666	1.233–5.765
	CMV positive	56	39.7	0.8110	1.072	0.606–1.896
Acute rejection episodes before PTLD	Present	62	44.0	0.0961	1.580	0.922–2.708
	Multiple episodes	23	16.3	*0.0073*	2.243	1.244–4.046
B-symptoms	Present	68	48.2	0.3214	1.326	0.759–2.316
	Absent	73	51.8			
ECOG status	0–2	128	90.8	*< .0001*	0.183	0.0910.368
	3–4	11	7.8			
Ann-Arbor stage	I	43	30.5			
	II	24	17.0			
	III	14	9.9			
	IV	55	39.0			
	Unknown	5	3.5			
	Limited stage (I–II)					
	Advanced stage (III–IV)			0.1997	1.447	0.823–2.543
Extra nodal disease	Yes	98	69.5	0.3704	1.345	0.703–2.570
	No	36	25.5			
	Unknown	7	5.0			
More than 1 extra-nodal site	Yes	32	22.7	0.1903	1.517	0.813–2.829
CNS	Yes	5	3.5	0.7181	1.240	0.386–3.979
Bone marrow	Yes	13	9.2	*0.0423*	2.184	1.027–4.643
Allograft	Yes	15	10.6	0.4536	1.355	0.612–3.001
GI tract	Yes	38	27	0.4692	0.788	0.414–1.501
Albumin at diagnosis	Normal	67	47.5	0.4515	0.809	0.466–1.405
	Low	65	46.1			
	Unknown	9	6.4			
CD20 status	Positive	104	73.8	0.9303	1.039	0.438–2.464
	Negative	18	12.8			
	Unknown	19	13.5			
Tumor EBER status	Positive	78	55.3	0.8760	0.954	0.531–1.714
	Negative	49	34.8			
	Unknown	14	9.9			
IPI score	0–2	84	59.6			
	3–5	41	29.1	*0.0422*	1.843	1.022–3.324
	Unknown	16	11.3			

Statistically significant P values (< 0.05) are shown in italics

#### Prognostic impact

Age at the time of transplant showed pediatric population has a better prognosis with HR: 0.207 (p < 0.0001 CI 0.105–0.407). Among adults, patients between 18–64 years had HR of 3.316 (p < 0.0001, CI 1.836–5.990) while elderly patients above 65 years of age had HR of 2.573 (p 2.573, CI 1.012–6.545).

Allograft type was also a statistically significant prognostic marker with lung transplant recipients having HR of 4.380 (p < 0.0001, CI 2.327–8.245) and liver transplant recipients had HR of 0.357 (p: 0.0023, CI 0.184–0.693) as compared to the rest of group. BMT patients also did poorly with HR of 2.197 (p: 0.0746, CI 0.925–5.219).

### EBV and CMV status

At the time of transplant, out of 141 transplant recipients, 58.2% (n = 82) were EBV seropositive while by the time of PTLD diagnosis 72.3% (n = 102) patients were seropositive for EBV. EBV serostatus of donors was mostly unavailable in records. Pathologic evaluation of tumor specimen for EBV showed, 56.0% (n = 79) were positive for EBV, 34.0% (n = 48) were negative and in 9.9% (n = 14) this information was not available. Epstein-Barr encoding region (EBER) in situ hybridization technique was used to identify EBV in the tissue specimen.

At time of transplant, CMV serostatus of recipients was positive for 39.7% (n = 56), negative for 52.5% (n = 74) and unknown for 7.8% (n = 11). CMV serostatus of donor was positive in 48.2% (n = 68), negative in 29.8% (n = 42) and was unknown for 22.0% (n = 31) patients.

#### Prognostic impact

The only significant poor prognostic factor was positive recipient EBV status with HR of 2.666 (p: 0.0127, CI 1.233–5.765). CMV status of either recipient or donor and the tumor EBER status were not significant.

### Immunosuppression and rejection episodes

Induction immunosuppression for transplant were used in 30.5% (n = 43) patients. Prior to diagnosis of PTLD, 44.0% (n = 62) had history of acute allograft rejection and 16.3% (n = 23) had more than 1 episode of rejection. Chronic allograft rejection was noted in 9.2% (n = 13) patients. At the time of PTLD diagnosis, tacrolimus was the most common immunosuppressant used either alone or in combination (63.1%, n = 89).

Prognostic impact: Patient with history of single or multiple episodes of acute rejections were noted to have HR of 1.58 (p: 0.0961, CI 0.922–2.708) while patients with multiple episodes of acute rejections had statistically significant HR of 2.2 (p = 0.0073, CI 1.244–4.046). Chronic rejection had a trend of poor prognosis but was not statistically significant. Induction immunosuppression was not found to be significantly associated with OS.

Use of tacrolimus at the time of diagnosis either alone or in combination with other immunosuppression for maintenance immunosuppression had a good prognostic impact with HR of 0.476 (p: 0.0068, CI 0.278–0.815). Similarly, use of azathioprine showed good prognostic impact with a p-value of 0.0558, almost statistically significant (HR 1.719, CI 0.987–2.994).

### Presenting characteristics

Median time from transplant to the diagnosis of PTLD was 4.1 years (range: 32 days–18.4 years). Early PTLD, defined as diagnosed within 1 year was noted in 24.8% (n = 35) patients while late-onset PTLD, which is defined as occurring after 1 year, was noted in 75.2% (n = 106) patients.

B-symptoms were present in 48.2% patients (n = 68) and fever was the most common B symptom noted in 39.0% (n = 55) patients. 90.8% (n = 128) had Eastern Cooperative Oncology Group (ECOG) performance status of equal or less than two.

CD20 status was positive in 73.8% (n = 104). Extra-nodal site was involved in 69.5% (n = 98) and most common extra nodal site GI tract (27.0%, n = 38). More than one extra-nodal site was involved in 22.7% (n = 32) and bulky disease (> 10 cm) was noted in 14.2% (n = 20) patients.

Prognostic impact: Early or late PTLD onset did not impact OS. The presence of B-symptoms was also not significant as well. Good performance status of ECOG 0–2 was a good prognostic factor with HR of 0.183 (p < 0.0001, CI 0.091–0.368). Bone marrow involvement had a poor prognosis with HR of 2.184 (p: 0.0423, CI 1.027–4.643). No other extra nodal site including CNS or allograft was found to have statistically significant hazard ratio. Other factors including serum LDH and albumin, CD20 status and bulky disease were also not significant.

### IPI score

IPI score was calculated in all 141 patients, but few patients did not have all required information to calculate IPI. Since missing information could have impacted actual IPI score, we excluded 16 patients where 1 or more item was missing from calculating the exact score. Of the remaining 125 patients, median IP score was 2 (Range 0–5).

Prognostic impact: Higher IPI score of 3–5 was found to have statistically significant HR of 1.843 (p: 0.0422, CI 1.022–3.324).

### Pathology

Table 2 shows the detailed pathologic distribution of study population along with HR of each sub-groups. DLBCL was noted to be the most common sub-type (42.6%, n = 60).

**Table 2:** Pathologic distribution, with HR

PTLD Pathology	Sub-types	B-cell subtype	*n*	%	p-value	Hazard ratios	95% confidence limits
Early lesion PTLD			11	7.8	0.9859	0.000	0.000–0.000
	Plasmacytic hyperplasia PTLD		4	2.8			
	Infectious mononucleosis PTLD		3	2.1			
	Florid follicular hyperplasia PTLD		4	2.8			
Polymorphic PTLD			27	19.1	0.6011	0.832	0.417–1.660
Monomorphic PTLD (B- and T-/NK-cell types)			97	68.8	*0.0177*	2.309	1.156–4.609
	B-cell		89	63.1	0.6285	0.790	0.304–2.054
		DLBCL	60	42.6	0.2096	1.413	0.823–2.426
		Burkitt	9	6.4			
		Plasma cell myeloma/Plasmacytoma like lesion	6	4.3			
		Follicular	2	1.4			
		Other B-cell type	2	1.4			
		Unspecified	10	7.1			
	T-/NK-cell		8	5.7			
Classical Hodgkin lymphoma PTLD			5	3.5	0.9852	0.000	0.000–0.000
Unknown			1	0.7			

Statistically significant P value (< 0.05) is shown in italics

Prognostic impact: Monomorphic PTLD had HR of 2.309 (p: 0.0177, CI 1.156–4.609). T cell PTLD or DLBCL did not have statistically significant impact on OS.

### Treatment

Out of 141 patients, 4.3% (n = 6) patients did not receive any treatment as either patient declined treatment or were not considered to be candidates for therapy. Immunosuppression was reduced (RIS) in 77.3% (n = 109) patients, as the only measure or in combination with other treatment modalities. The upfront treatment approach is outlined in Table 3.

**Table 3:** Upfront therapy with hazard ratio

PTLD group/sub-group	Upfront therapy	*n*	*%*	p-value	Hazard ratios	95% confidence limits
Cumulative	RIS alone	33	23.4	0.1454	0.589	0.289–1.201
	Rituximab (± RIS)	35	24.8	*0.0309*	1.930	1.062–3.505
	Chemotherapy (± RIS, ± rituximab)	65	46.1	0.6196	0.865	0.487–1.535
Monomorphic PTLD	RIS alone	12	13.19	0.7359	1.163	0.484–2.793
	Rituximab (± RIS)	25	27.47	0.2383	1.502	0.764–2.953
	Chemotherapy (± RIS, ± rituximab)	54	59.34	0.1939	0.651	0.341–1.244
Early lesions and polymorphic PTLD	RIS alone	19	52.78	0.1545	0.396	0.111–1.417
	Rituximab (± RIS)	10	27.78	*0.0498*	3.765	1.001–14.161
	Chemotherapy (± RIS, ± rituximab)	7	19.44	0.9151	0.919	0.193–4.374

Statistically significant P values (< 0.05) are shown in italics

**Fig. 4:**
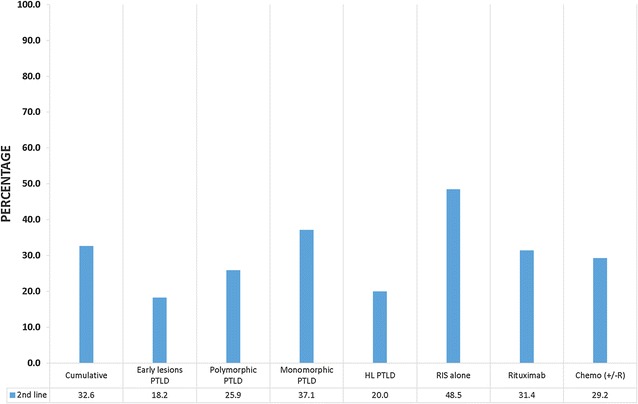
Comparison of patients needing further lines of therapy

**Fig. 5:**
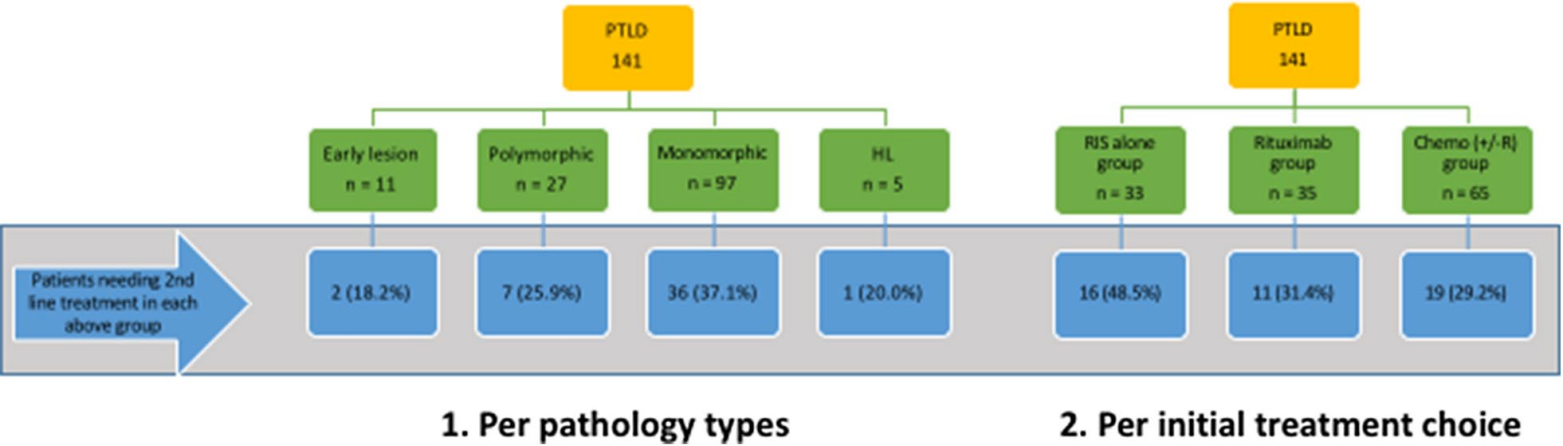
Flow chart showing need of 2nd line treatment based on pathology type and initial treatment choice

After initial or upfront therapy, 32.6% (n = 46) patients needed 2nd line therapy, and another 9.2% (n = 13) needed third line therapy. Subsequent therapy was needed for patients who either progressed or relapsed after first line therapy and rarely for intolerance. At the end of the study, 60.3% (n = 85) eventually needed chemotherapy with or without rituximab as part of either first line, second line, or third line therapy. CHOP (± R) was the most common regimen used and was used in 28.4% (n = 40) of overall patients. Figure 4 shows patients in different groups needing second-line therapy as compared to the cumulative. 48.5% of patients treated with RIS alone as the first option needed further therapy. Similarly of all patients with monomorphic PTLD and treated with RIS alone, 37.1% of them needed further therapy. Table 4 shows distribution of different chemotherapeutic regimens as first, second and third line therapy. Figure 5 shows patient distribution needing 2nd line treatment based on pathology type and initial treatment choice.

**Table 4:** Details for rituximab and chemotherapy regimens used during overall treatment

Therapy type	Sub-type	As 1st line therapy		As 2nd line therapy		As 3rd line therapy	
		n	%	n	%	n	%
Rituximab alone		35	24.8	4	2.8	2	1.4
Chemotherapy (+ R)	R-chop	22	15.6	11	7.8	2	1.4
	R/C (cyclophosphamide)	20	14.2	5	3.5		
	R-EPOCH	2	1.4				
	R-CVP	4	2.8	2	1.4	1	0.7
	BR	1	0.7	1	0.7		
	R-hyperCVAD	1	0.7				
	RICE	1	0.7	1	0.7		
	R-Mtx	1	0.7				
Chemotherapy (− R)	CHOP	5	3.5	1	0.7		
	ABVD	1	0.7	1	0.7		
	CVP	1	0.7	1	0.7		
	CEOP	1	0.7				
	Other regimens	5	3.5	12	8.5	8	5.7

Prognostic impact: Surprisingly, patients treated with Rituximab alone as front line therapy with or without RIS did poorly with HR of 1.930 (p: 0.0309, CI 1.062–3.505). On subgroup analysis, this was again statistically significant in less aggressive PTLD group comprising of early lesions and polymorphic types of PTLD with HR of 3.765 (p: 0.0498, CI 1.001–14.161), but was not significant for monomorphic PTLD group.

Use of rituximab anytime during treatment either alone or with chemotherapy did not have statistically significant impact on OS and similarly use of CHOP any time during treatment was not statistically significant for OS, as compared to the rest of group.

### Response

At the end of the study, 62.4% (n = 88) had complete response while 18.4% (n = 26) had progressive disease. ORR was 68.1% (n = 96) while DCR was 74.5% (n = 105). Table 5 shows patient outcomes at the end of the study.

**Table 5:** Outcomes of patients after PTLD diagnosis and treatment

Outcome measures	Final outcome	
	n	%
CR	88	62.4
PR	8	5.7
SD	9	6.4
PD	26	18.4
Unknown	10	7.1
DCR	105	74.5
ORR	96	68.1
Alive	81	57.4
Deceased	56	39.7
Unknown	4	2.8
Alive without PTLD	69	48.9
Alive with PTLD	12	8.5
Graft survived	116	82.3
Graft failed	17	12.1
Graft status unknown status	8	5.7

57.4% (n = 81) patients were still alive at the end of study. Of these, 48.9% (n = 69) are alive without PTLD while 8.5% (n = 12) are alive with PTLD. 39.7% (n = 56) patients are deceased and status of 2.8% (n = 4) is unknown. Of deceased patients, 17.7% (n = 25) died due to PTLD or treatment related complications, 14.2% (n = 20) died of unrelated reasons while cause of death in 7.8% (n = 11) in not available.

#### Prognostic impact

After any kind of first-line therapy, patients who achieved CR had HR of 0.356 (p: 0.0006, CI 0.198–0.641) while patients who had PD had HR of 6.130 (p < 0.0001, CI 3.354–11.204).

#### Outcome based on PTLD subtypes

**Fig. 6:**
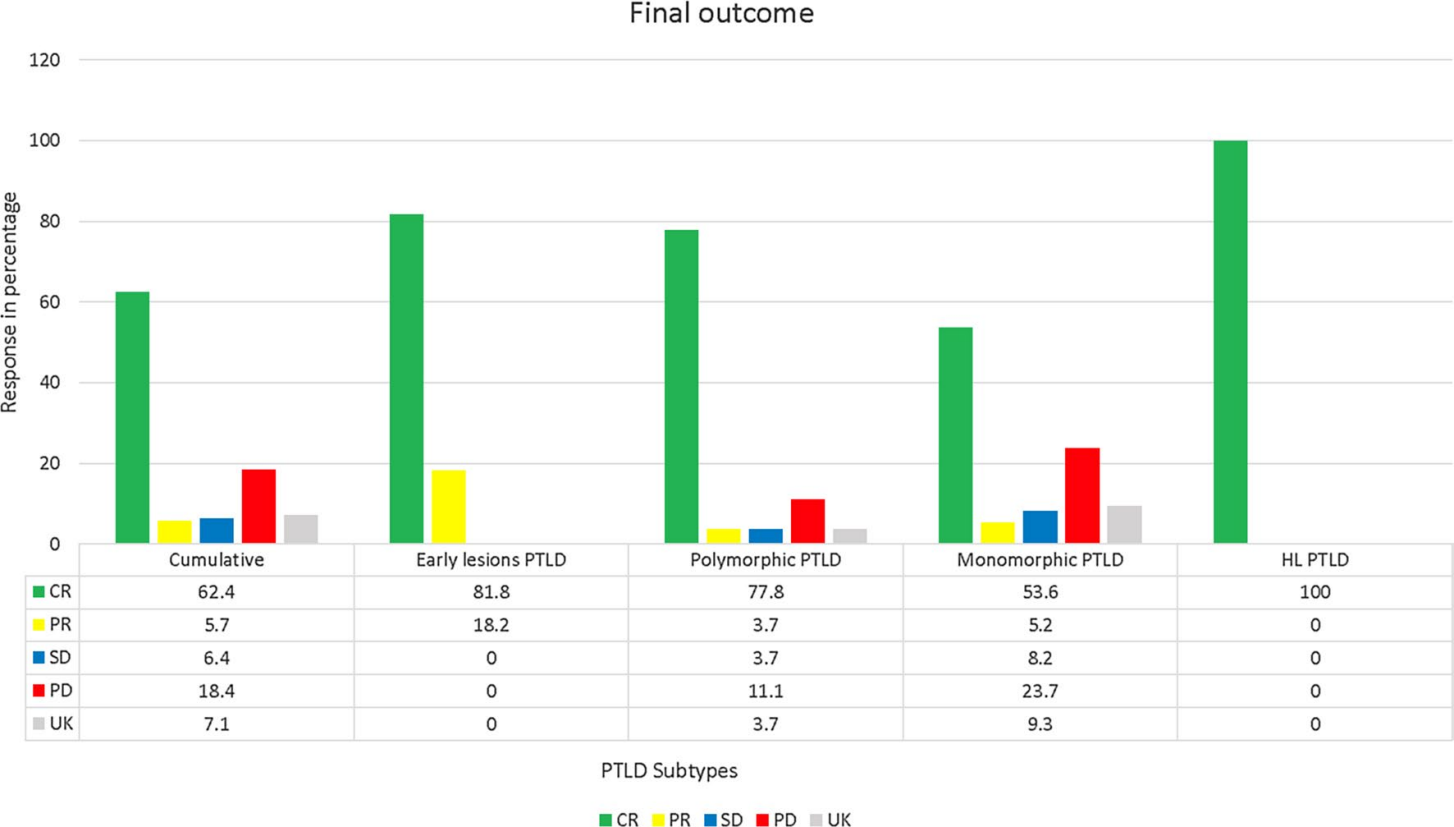
Shows final outcome of different PTLD types as compared to cumulative

Figure 6 shows distribution of final outcomes of different PTLD types as compared to cumulative group.

### Graft outcome

After PTLD diagnosis, grafts survived in 82.3% (n = 116) and failed in 11.3% (n = 16) patients. During PTLD treatment 18.4% (n = 26) experienced treatment related graft rejection which led to graft failure in 4.3% (n = 6) patients while graft in 14.2% (n = 20) patient was able to be salvaged.

#### Prognostic impact

Rejection episodes during PTLD treatment did not impact OS.

## Discussion

This retrospective study from a regional transplant center of South-East USA has a good sample size which is among one of the largest reported studies so far throughout the world. Our study identified multiple statistically significant prognostic factors which do impact overall survival and can help in establishing guidelines for further therapy of this rare disease. Table 6 shows comparative results of prognostic factors identified in previous major studies. We compared our results with findings of these studies.

**Table 6:** Comparative results of statistically significant (p < 0.05) prognostic factors in major studies reported worldwide and compared to this study

Study	Leblond et al. [23]	Jain et al. [24]	Ghobrial et al. [25]	Maecker et al. [26]	Oton et al. [27]	Knight et al. [28]	Evens et al. [29]	Kinch et al. [30]	Dierickx et al. [31]	Montanari et al. [32]	Luskin et al. [33]	This study
Year	2001	2002	2005	2007	2008	2009	2010	2013	2013	2015	2015	2017
Subjects *(n)*	61	170	107	55	84	78	80	135	140	120	176	141
Advanced age at diagnosis	NSS	NSS	SS					SS	SS	SS	SS	SS
Performance status	SS		SS		SS	NSS	SS	SS	SS	SS		SS
Late PTLD onset	Poor (NSS)	NSS	NSS	NSS	Good (SS)		NSS		NSS	NSS		NSS
Gender	NSS	F > M (NSS)	NSS					NSS		NSS		F > M (SS)
Extra-nodal site	SS		SS	SS	SS	NSS	SS	SS	SS	NSS	SS	SS
CD20 +			NSS						NSS	SS		NSS
Low albumin							SS		SS	NSS		NSS
High LDH	NSS		SS				NSS	SS	SS	NSS	SS	NSS
B-symptoms						NSS		SS			NSS	NSS
Allograft type	NSS		NSS	NSS				SS		NSS	SS	SS
Non EBV PTLD	SS	NSS		NS				SS		NSS	NSS	NSS
Advanced stage	NS		SS	SS	SS	SS	NSS	SS	SS	NSS	SS	NSS
PTLD type/grade	NSS	SS		NSS					SS	NSS	NSS	SS
Higher IPI	NSS		SS			SS	NSS		SS			SS
Initial response			SS	SS								SS
Rituximab use			NSS		SS		SS			NSS		NSS

SS statistically significant, *NSS* not statistically significant

Certain prognostic factors are well known including age, performance status, extra-nodal site involvement, low albumin and IPI score. These findings represent age, extent and nutritional status which is expected to impact survival. In contrast to these known factors, albumin was not significant in our study.

We found that female gender to have statistically significant better prognosis which was also noted by Jain et al. but was not statistically significant in that study [24]. The advanced stage of PTLD was not significant, but in PTLD types monomorphic PTLD did perform poorly which is known to be an aggressive form of PTLD with poor prognosis. T-cell PTLD was found to do poorly in some studies but not in our study [23, 30].

The role of EBV is very well known in the pathogenesis of PTLD, but its impact on survival is still not clear. EBV positivity was reported to be a poor prognostic factor by Oton et al. [27] while non-EBV PTLD was seen as poor prognostic by Leblond et al. [23] and Kinch et al. [30]. Our study did find that EBV recipients tend to do poorly, but EBER positive PTLD had no significant impact on survival. These findings do support the role of EBV in the pathogenesis of PTLD, but its impact on survival needs to be studied further.

As seen in our study, the initial response to treatment has been seen as a prognostic value in studies done by Ghobrial et al. [25] and Maecker et al. [26]. This does show that patients who do not respond with early treatment options should be managed considering that they may have poor survival as compared to the rest of group.

Patients who were on tacrolimus and azathioprine for maintenance immunosuppression at the time of PTLD diagnosis performed better in this study and similar finding for tacrolimus was noted by Jain et al. [24], although interestingly previous studies have also shown increased incidence of PTLD with tacrolimus use [7, 34]. The significance of allograft rejection episodes in PTLD incidence is well known [35, 36] but we also noticed that patients who had acute rejections, especially multiple rejections performed poorly, and similar findings were noted by Kremers et al. [35]. Transplant recipients who experience episodes of rejection often get increased immune-suppression for allograft protection. Our study findings signify the role of chronic immunomodulation over the years for these patients and need to be considered while selecting chronic immune suppressants and during rejection treatments. As noted in our study, patients who experience rejections and later on develop PTLD will tend to do poorly. We do recommend close follow up with immunologists or transplant teams who can navigate these patients through these complexities of immunosuppression management.

Rituximab use was seen statistically significant related with good prognosis by Oton et al. [27] and Evens et al. [29]. but not in other studies [25, 32]. In our study rituximab use was not associated with improved survival as compared to the rest of group. Strikingly in our study, we noticed that isolated use of Rituximab as upfront therapy had poor hazard ratio in the cumulative group as well less aggressive subgroup comprising early lesion and polymorphic PTLD. We could not identify any factor which could explain this finding as patients who received Rituximab alone had comparable performance status and disease status to rest of group. They had a poor initial response which in itself is a poor prognostic finding in our study. These findings support further studies looking into the role of rituximab in PTLD, especially isolated use without other chemotherapeutic agents. Although CHOP (± R) was noted to be the most common regimen used, we did not find any statistically significant results of CHOP use.

### Study limitations

This study was done as a retrospective study, and the main aim of this study was to identify new and validate previously known prognostic factors. Authors do acknowledge the inherent limitations of a retrospective study, but we do feel that good sample size of this study acquired over prolonged period does make results reliable. Most of the PTLD treatment guidelines are derived from retrospective studies from different transplant centers, like our study and with contribution from limited prospective trials due to the rarity of this disease [16, 20]. Treatment regimens and dosages used to treat PTLD were as per the current guidelines as per discretion of treating physicians. This study goes back over a prolonged period when EBV PCR was not routinely available or monitored as the standard of care. This value was not available for many patients, especially in the adult population. Also, this study was not focused on epidemiology or incidence of PTLD.

## Conclusion

This study, which is one of the largest studies done so far has validated some of the long-established prognostic factors while identifying some new factors and challenging others. We hope this study will further help physicians in making treatment choices based on these prognostic factors.

## Abbreviations

PTLD: post-transplant lymphoproliferative disorder
IRB: institutional review board
WHO: World Health Organization
SOT: solid organ transplant
HSCT: hematopoietic stem cell transplant
DLBCL: diffuse large B cell lymphoma
EBV: Epstein-Barr virus
RIS: reduced Immunosuppression
UF: University of Florida
R-CHOP: rituximab, cyclophosphamide, doxorubicin, vincristine and prednisolone
RCP: rituximab, cyclophosphamide, and prednisone
OS: overall survival
CR: complete remission
PR: partial remission
SD: stable disease
PD: progressive disease
DCR: disease control rate
ORR: overall response rates
ECOG: Eastern Cooperative Oncology Group
CMV: cyto-megalo virus
HR: hazard ratio
EBER: epstein-barr encoding region
